# Same-sex love and marriage: understanding the Special Marriage Act, 1954

**DOI:** 10.3389/fsoc.2025.1622997

**Published:** 2026-01-09

**Authors:** Theodora Cecilia Gomes, Sruti Kanungo, Seonti Sengupta

**Affiliations:** Department of Humanities and Social Sciences, Indian Institute of Technology (Indian School of Mines), Dhanbad, India

**Keywords:** same-sex marriage, queer rights, Special Marriage Act, LGBTQ, homosexuality

## 1 Introduction

“*Marriage is a social institution. Marital status is not conferred by the state. The idea of marriage is not a fundamental right”*—Justice Ravindra Bhat (while passing the Supriyo Chakraborty v. Union of India judgment on the petitions filed for legalizing same-sex marriages in India on 17th October 2023).

Marriage, as a social institution, has been shaped by traditional heterosexual social norms. The society treats “heterosexuality” as normal and any sexual act other than this is deviant and illegal (Rich, 1980; Srivastava, 2014; Goldfarb, 1997; Bartholomay, 2018). However, in the recent decade, there has been a rising debate on same-sex marriages globally. While western nations like the USA, UK, Canada, Australia, Switzerland, Germany, and Thailand, have achieved marriage equality, legalizing same-sex marriage in the Indian context has become essential to promote inclusion and equality. Advocates of queer rights in India call for the inclusion of same-sex couples in the Special Marriage Act (1954), to grant them marriage equality.

This paper attempts to understand why Special Marriage Act (1954), cannot accommodate same-sex couples. The paper proceeds in three parts. The first section describes the history of homosexuality and law in India. The second section analyzes the Special Marriage Act (1954), and its heteronormative limitations. In the final section, the authors offer alternative judicial frameworks to legalize queer marriages in India.

## 2 Homosexuality in ancient and colonial India

Ancient and medieval inscriptions, texts, and paintings have acknowledged the existence of homosexuality in India. In the Mahabharata,^1^ Princess Shikhandini,^2^ the daughter of King Draupad, was a transman who defeated Bhisma with the help of a “Yaksha” or “Nature spirit” in Ancient Indian mythology (Doniger, 2011). The Kamasutra^3^ depicts instances of homosexuality, such as the presence of transgenders or “*Tritiya Prakriti,”* and oral sexual activities or “*Auparistaka”* among men and women. The Khajuraho temple^4^ in Madhya Pradesh also portrays sculptures engaged in homosexual sex. The 17th-century poem “*Haqiqat al Fuqura*” was written about two homosexual inter-religious lovers, Hussayn and Madho (Kidwai and Vanita, 2001).

During the colonial period, queer lives were disrupted due to British values of sexuality. They saw homosexuality as a “crime” or “sin,” which was against the law of nature. Victorian morality was built on hyper-masculine principles that ignored same-sex desire and gender non-conformity (Saxena and Mahajan, 2020). The Indian Penal Code (IPC) was introduced in 1860 by Lord Macaulay, the President of the Indian Law Commission. Chapter XVI of IPC, Section 377, read^5^

“*377. Unnatural Offenses - Whoever voluntarily has carnal intercourse against the order of nature with any man, woman, or animal shall be punished with imprisonment for life or with imprisonment of either description for a term which may extend to ten years, and shall also be liable to fine.”*

Section 377 did not acknowledge “consent” during sexual intercourse, thus equating homosexual sex with rape. Foucault discusses “disciplined bodies” under the state's gaze and how they serve as a form of disciplinary control. This reiterates the colonial mindset that queer individuals need to be regulated as they are seen as criminals (Goodman, 2001). Foucault's statement “*a state of conscious and permanent visibility that assures the automatic functioning of power*” reflects the above (Foucault, 2012).

The first case registered under Section 377 in India was in 1884 in the Queen Empress v. Khairati case.^6^ The police arrested Khairati, a transgender person from Moradabad district, without any complaint, simply because she was cross-dressing and singing with a group of women in her village (Narrain, 2018). She was labeled as a “habitual sodomite” upon close medical examination. In India, around 200 such cases have been registered under Section 377 since its implementation.^7^

## 3 Homosexuality in contemporary India

The year 2018 marked an important milestone in the history of LGBTQ rights in India as Section 377 of the IPC was scrapped, thereby decriminalizing homosexuality. The Supreme Court, led by a five-judge bench comprising Chief Justice Misra, Justice Khanwilkar, Justice Nariman, Justice Chandrachud, and Justice Malhotra, ruled unanimously in Navtej Singh Johar v. Union of India^8^ that Section 377 was unconstitutional because “*it criminalizes consensual sexual conduct between adults of the same sex.”* This victory was a product of the two-decade-long struggles of the NAZ foundation, which was the first NGO to file a Public Interest Litigation^9^ before the Delhi High Court in 2001 to legalize homosexual intercourse between two consenting adults. While delivering this verdict, the Supreme Court had extensively referred to the NALSA judgment^10^ (2014) and the Yogyakarta principles^11^ (2006) and stated that the Yogyakarta principles “*conform to our constitutional view of the fundamental rights of the citizens of India and persons who come to this Court”* (Paragraph 84, Justice Indu Malhotra). After the decriminalization of homosexuality in India, the next step was to fight for legal recognition of same-sex marriages.

## 4 India's plural legal system

India has a plural legal system, where multiple forms of law coexist. India is mainly governed by three types of laws: formal, customary, and personal law. Formal state laws are the Indian Penal Code, the Constitution, Statutes, and various Supreme Court and High Court judgments. Customary laws are norms based on caste,^12^ kinship systems, and *khap panchayats*.^13^ Personal or religious laws include the Hindu, Muslim, and Christian laws governing marriage, inheritance, and family. Although Section 377 has been decriminalized by the state, queer identities are controlled by personal and customary laws.

In India, personal and customary laws are based on traditional regulatory measures. For example, the Hindu Marriage Act (1955), defines marriage as a union between a “bride and bridegroom” (Section 5). So, there is no scope for queer couples. Further, the Hindu Succession Act (1956), denotes that succession must be maintained through patrilineal descent. It assumes family membership through heterosexual marriage and children by marriage/adoption. Terms such as “husband,” “wife,” “widow,” “son,” and “daughter” are used^14^. There is no mention of queer spouses or non-binary individuals, and they can't claim inheritance. Additionally, Muslim marriages are governed by the Muslim Personal Law (Shariat) Application Act, 1937, which states that marriage or “*Nikaah”* is a contract between a “man” and a “woman,” excluding queer couples. Christians in India marry under the Indian Christian Marriage Act (1872), which describes marriage as a sacrament between a “man” (above 21) and a “woman” (above 18), that must be solemnized by a licensed minister. Indian Christians do not have a separate law for inheritance. They are governed by the Indian Succession Act (1925). Queer partners cannot inherit property, except through private wills.

India has followed a system of legal pluralism since the colonial period. Due to diverse communal differences, the British found it difficult to establish a single codified law to govern the country. Instead, they formalized individual customs, rules, and norms of these religious communities in personal laws. Unlike Western countries, India does not follow a uniform civil code.

## 5 Special Marriage Act, 1954

The Special Marriage Act was enacted in 1954 to provide an alternative to these individual personal laws. This act was created to facilitate inter-caste and inter-religious marriages that were previously prohibited under their personal laws. Couples undergo a civil registration process, and their issues related to marriage, divorce, and succession are governed by a secular state law. The SMA is one of the most progressive and secular laws in India, as it allows individuals to marry partners of their own choice, free from the restrictions of caste, religion, and community.

Queer activists in India argue for the inclusion of queer couples in the SMA because it offers a non-religious secular framework. However, this is not structurally feasible because the Act's language explicitly mentions gendered terms like “male” and “female” and heteronormative categories like “husband” and “wife.”

Section 4 (c) of SMA states that a marriage is legally valid if “*the male has completed the age of twenty-one years and the female the age of eighteen years.”* The verbatim of this section clearly reveals that this law does not cater to same-sex couples.

Two petitions were filed in 2022 to challenge the provisions of the SMA. Supriyo Chakraborty and Abhay Da,^15^ a gay couple filed a petition at the Delhi High Court, and Adhila Nasrin filed a habeas Corpus Writ petition in the Kerala High Court. The primary argument by the petitioners was to amend the SMA. Section 15 (a) of SMA states that a marriage can be registered if “*a ceremony of marriage has been performed between the parties and they have been living together as husband and wife ever since.”* The petitioners argued that the terms “husband” and “wife” should be modified to “spouse” or “partner” to set a gender-neutral tone.

## 6 Decoding Supriyo v. Union of India

In response to petitions demanding the legalization of same-sex marriages, a five-judge constitution bench was formed in 2022 by the Supreme Court of India, comprising Chief Justice D.Y. Chandrachud, Justice S.R. Bhat, Justice S.K. Kaul, Justice P.S. Narasimha, and Justice Hima Kohli. The final verdict, passed on October 17, 2023, had two contradictory sides with different perspectives. The majority opinion was composed of Justice S.R. Bhat, Justice P.S. Narasimha, and Justice Hima Kohli. The minority opinion was composed of Chief Justice of India (CJI) D.Y. Chandrachud and Justice S.K. Kaul.

The minority opinion emphasized that homosexuality is native to India and not a product of Westernization. Transgenders have always existed in Indian society, known as *hijras*,^16^ *kothis*,^17^ *aravanis*,^18^ etc., (Reddy, 2005). Queerness is also not an “elite or urban” concept, as various petitioners came from rural areas such as Muktsar (Punjab), Durgapur (West Bengal), and Vadodara (Gujarat). The visibility of queer communities is more in urban spaces due to the anonymity offered by metro cities. The idea of marriage is dynamic, and issues such as sati,^19^ widow remarriage, child marriage, dowry,^20^ inter-caste marriage and divorce have evolved with time.

In contrast, the majority view opined that granting legal validity to queer marriages can only be done by the Parliament. The Supreme Court does not have the power to enact such laws or create a regulatory framework. However, they reiterate that transgender individuals are allowed to get married in India as per the NALSA judgment of 2014.^21^ If a transgender person is in a heterosexual relationship, she/he is free to marry her/his partner as the Indian laws only recognize marriage between a “man” and “woman,” or a “bride” and “groom.”

## 7 Complexity of Indian family laws and constitutional

Marriage is a central aspect of family law in India. Laws such as inheritance, adoption, and divorce are all tied to it. Criminal laws are very specific in nature. Therefore, clear and concise definitions are required regarding same-sex marriage. For example, there are certain laws that only punish the husband and his family members. In case of Domestic Violence, which was punishable under 498A of Indian Penal Code,^22^ corresponding to Section 85 and 86 of Bharatiya Nyaya Sanhita (BNS),^23^ specifically uses the word “husband.” The verbatim of Section 85 BNS starts with “*Husband or Relative of Husband of woman subjecting her to cruelty…”*. It is difficult to differentiate between a “husband” and “wife” in a same-sex relationship. Butler's lens of “*gender performativity,”* would view gender as a social construct, rather than a biological disposition (Butler, 1990). The use of rigid categories, by legislative institutions, such as “husband,” “wife,” “male,” and “female,” produces and reproduces the binaries of gender. When legal language uses these terms, it reinforces heteronormativity and legally excludes the experiences of transgender individuals, non-binary, and queer people. Therefore, without first making gender neutral laws, queer marriages cannot be regularized and legalized.

The final verdict on the Supriyo Chakraborty V Union of India (2023) considered three major factors before passing the judgment. (a) Tushar Mehta, the Solicitor General of India, representing the state, opined that due to the separation of the three powers, only the Parliament could enact laws, not the Apex Court (Sharma, 1980). Prior to the Supriyo judgement, the Bar Council of India (BCI) passed a resolution on 23rd April 2023, stating that “*99.9 % of Indians are against same-sex marriage,”* so the Supreme Court should let the legislature make this decision. This data was verified as a false claim^24^ by Boom Live. (b) It is not possible to amend the SMA to make provisions for homosexual marriages because it will lead to changes in other interconnected laws^25^ that are regulated by the state, such as the age of consent, divorce, succession, and adoption. (c) Marriage in India has religious and socio-cultural connotations and falls under personal laws (Ruwali, 2018). A survey by CSDS Lokniti and Azim Premji University (2019)^26^ found out that more than 50% of its respondents do not approve of same-sex relationships due to the conservative mindset. Interfering with personal laws can cause religious backlash. For example, the communal riots due to Mohd Ahmed Khan V Shah Bano Begum^27^ case, which stated a Muslim woman's right to maintenance under Section 125 of the Code of Criminal Procedure 1973, which is outside the Muslim personal law.

From the sociological standpoint the Special Marriage Act cannot accommodate same-sex marriages righteously because it does not recognize the broader horizon of Indian Society. Caste forms the foundation of the Indian culture. It holds significance in maintaining the social homeostasis. Caste system adheres to endogamy^28^, a practice that perpetuates indigenous values and customs through the reproduction of families (Dumont, 1980). However, in recent years, there has been a growing trend of inter-caste marriages in India. These marriages are being hailed as progressive acts.

Homosexual desires transgress hetero-patriarchy as well as Dumont's idea of purity and pollution. Queer Dalit lovers face double oppression due to their caste and gender non-conformity status. Liberation of homosexuality is not possible without the annihilation of caste (Ambedkar, 1936).

## 8 Judicial alternatives to the Special Marriage Act, 1954

Since SMA can't be modified, we offer three suggestions by which queer marriages can be legalized in India.

(i) The most vital step to legalize same-sex marriage is to reform personal laws. Failure to enact a comprehensive uniform civil code shows the significant importance of personal laws in India. Existing marriage laws must be reinterpreted to include queer couples (Agarwal, 2022). Laws must be created for same-sex intimate partner violence and recognition of non-kin relationships among the hijra community, such as teacher-disciple, mother-daughter, and husband-wife.
(ii) Marriage rights can be extended to the community by drafting a new Act and treating the LGBTQ population as a separate entity. India can follow the USA's footsteps, like the Respect for Marriage Act, which was enacted on December 13, 2022, replacing the Defense of Marriage Act (DOMA). However, this would require approval from the Indian parliament, which is unlikely due to constitutional challenges and opposition from political and religious groups.
(iii) Most importantly, the government can attribute a different status, such as “civil partnership” or “cohabitants,” to queer couples where they can claim equal benefits like heterosexual couples, such as opening joint bank accounts, claiming insurance money, filing tax returns together, etc. A survey by IPSOS (2021)^29^ found that nearly 14% of Indians support same-sex civil unions. Civil Union is available in various countries, such as the United Kingdom, under the Civil Partnership Act of 2004, and Italy, under the Civil Union Law, enacted on June 5, 2016. This perspective is also supported by CJI Chandrachud and Justice Kaul, who stated that queer couples already have cohabitation rights, as per Articles 19 and 20 of the Indian Constitution and the Puttaswamy judgement (2017), and should be granted “civil union” status.^30^ Among the three alternatives, the most feasible option is to allow same-sex couples to register their “civil union” as an interim step and gradually work toward achieving marriage rights through future litigation.

## 9 Conclusion

On 1st July 2024, the Indian Penal Code (IPC) was replaced by the Bharatiya Nyaya Sanhita^31^ (BNS). The IPC was established in 1860 under British colonial law. BNS was proposed in 2023 to modernize the archaic law and address contemporary issues in independent India. A major limitation of the BNS is the removal of Section 377, which criminalized rape against non-minor males and animals (bestiality). Currently, there is no legal framework to address sexual assault against men. With regard to the LGBTQ community, the laws in BNS must be created in a gender-neutral way so that they can be applied to same-sex couples without any complications.

Although the Supreme Court has not recognized same-sex marriages in its latest judgment (Supriyo Chakraborty v. Union of India, 2023), the Constitution must be accommodative of the fundamental rights of queer individuals. A survey conducted by the Pew Research Center^32^ (2023) found that 53% of Indians favor same-sex marriages. Legal marriage not only grants social approval but also provides the practical benefits of being a spouse, such as access to medical emergencies, financial and banking matters, inheritance, succession, and adoption.

Modifying the Special Marriage Act to incorporate same-sex couples is not feasible in the Indian context, as religion, class, and caste play a vital role in determining our life world. *Intersectionality* is an essential tool to understand the Indian LGBTQ community, because not all queer individuals experience oppression uniformly (Crenshaw, 1989). An upper-class gay man has more access to life chances and resources than a working-class Dalit lesbian woman. Mainstream queer activism in India is often criticized as it is controlled by urban upper class upper caste men, often ignoring the voices of Dalit, working-class, disabled, and rural individuals. These “*sexual subalterns”* are individuals whose identities are defined by “*loss,”* invisibility, and erasure in the history of sexuality in South Asia. The LGBTQ community is often silenced and misrepresented in global narratives and media. They are erased, not because of their marginal position, but because society refuse to acknowledge them. Homoerotic desire must be studied in “*abundance,”* to understand intersectional queer identities and heteronormativity of caste (Arondekar, 2023).

A review petition was filed on 1st November 2023, challenging the Supriyo judgment. However, it was rejected by the Supreme Court on 9th January 2025. As stated by Justice Indu Malhotra (2018), “*History owes an apology”* to the members of the LGBTQ community. It is not morally or legally acceptable to deprive a community of its rights simply because they form a “*minuscule fraction.”*

## Footnotes

1 Ancient Indian Hindu epic, describing the story of two cousins, the Kauravas and Pandavas, in a war of succession known as the Kurukshetra War.

2
https://www.indianmythologystories.com/2024/06/the-untold-story-of-mahabharata-mystery.html

3 Ancient Indian Hindu text, written in Sanskrit, that describes sexual positions, intimacy, and desire. Lesbian sex is mentioned in Chapters 5 and 8 in Book 2 of the Kamasutra.

4 The Khajuraho temple is located in Madhya Pradesh, which was built during the Chandela dynasty. It features erotic sculptures, including both heterosexual and homosexual couples engaged in sexual acts.

5
https://indiankanoon.org/doc/1836974/

6
https://www.himalmag.com/comment/transforming-the-constitution-transgender-persons-protection-rights-bill

7
https://indianexpress.com/article/india/section-377-timeline-homosexuality-gay-sex-5253129/

8 Navtej Singh Johar V Union of India, WP (c) 76/2016.

9 Naz Foundation V Govt of NCT of Delhi – WP (c) 7455/2001.

10 National Legal Services Authority (NALSA) vs. Union of India (2014) is a landmark judgement that recognized transgender individuals as the third gender under the Indian Constitution and provided a legal framework for their rights.

11 The Yogyakarta Principles are a document outlining 29 principles on LGBTQ rights, which were drafted and developed by the International Commission of Jurists and the International Service for Human Rights, on behalf of a coalition of human rights organizations, activists, and jurists. This was a product of a meeting held at Gadjah Mada University in Yogyakarta, Indonesia, from November 6 to 9, 2006.

12 Caste is a hierarchical division in Hindu society consisting of four layers, Brahmin, Kshatriya, Vaishya, Shudra, and a fifth group known as the ‘untouchables'.

13 Khap Panchayat is a form of rural government, present in Indian villages. They maintain harmony among different communities. Members are not elected, but rather selected based on caste and gotra (clan affiliation).

14 However over the time, Hindu Succession (Amendment) Act 2005 amended the Hindu Succession Act (1956), to eliminate gender discrimination by granting daughters equal rights as sons.

15 Supriyo Chakraborty V Union of India – WP (c) 1011/2022.

16 Hijra refers to the third gender community, present in South Asia. Some hijras are born intersex, others are born male, but dress up as female. Some undergo a castration ceremony and remove their male genitalia.

17 Kothi refers to a man who takes up an effeminate role in a same-sex relationship. Their active partner who penetrates is known as a panthi. Unlike hijras, kothis do not live in communal housing.

18 Aravanis are transgender individuals living in Koovagam, Tamil Nadu.

19 Sati is an abolished Hindu practice, which involved the self-immolation of a widow at her husband's pyre.

20 Dowry is an abolished Hindu practice, where wealth, property, and other valuable items are given to the groom's family by the bride during marriage.

21 Arunkumar V The Inspector General Of Registration – WP (c) 4125/2019, NALSA (Check footnote 11).

22 *Whoever, being the husband or the relative of the husband of a woman, subjects such woman to cruelty shall be punished with imprisonment for a term which may extend to three years and shall also be liable to fine*.

23 BNS, or Bharatiya Nyaya Sanhita of 2023, is India's new criminal law code, replacing the colonial-era Indian Penal Code (IPC) of 1860.

24
https://www.boomlive.in/fact-check/fact-check-viral-marriage-equality-same-sex-marriage-lgbtqia-lgbtq-bar-council-of-india-21804

25 “*Any alteration or substitution of words in SMA, as was suggested by the petitioners, would impact a string of provisions in other statutes, apart from impacting personal laws” –* Tushar Mehta (https://api.sci.gov.in/supremecourt/2022/36593/36593_2022_1_1501_47792_Judgement_17-Oct-2023.pdf). Acts like Hindu Marriage Act (1955); Hindu Succession Act (1956); Hindu Adoption and Maintenance Act, 1956; Juvenile Justice (Care and Protection of Children) Act, 2015; Central Civil Services (Pension) Rules, 1972; Indian Succession Act (1925) and others need to be reinterpreted because they use terms like ‘husband', ‘wife', ‘widow', ‘widower', ‘heir of husband' to establish laws, that are centered on a heterosexist marriage framework.

26
https://www.lokniti.org/media/upload_files/politics-and-society-between-elections-2019-report.pdf

27 Mohd Ahmed Khan v Shah Bano Begum 1985 SCR (3) 844, the Supreme Court granted alimony to a Muslim woman under secular law, which was seen as interference with Islamic personal law. Legalizing same-sex marriages is not feasible as it requires modifying personal laws. Key terms like ‘degrees of prohibited relationship', ‘restitution of conjugal rights', ‘judicial separation', ‘divorce', ‘permanent alimony and maintenance', ‘disposal of property' need to be defined in reference to same-sex couples.

28 Endogamy refers to marrying within one's own social group (class, caste, community, religion).

29
https://www.ipsos.com/en-in/significant-support-rights-lgbtq-among-urban-indians-ipsos-lgbtq-pride-2021-global-survey

30 Justice K. S. Puttaswamy (Retd.) Vs. Union of India, WP (c) 494 / 2012. The Supreme Court passed a landmark judgment in 2017, stating that the right to privacy is a fundamental right under Article 21 (personal liberty) of the Indian Constitution.

31
https://indiankanoon.org/doc/149679501/

32
https://www.pewresearch.org/short-reads/2023/11/27/how-people-around-the-world-view-same-sex-marriage/
